# The promising potential of camelid nanobodies for nuclear medicine

**DOI:** 10.1007/s00259-025-07136-y

**Published:** 2025-02-21

**Authors:** Hans J. Biersack, Alejandro Rojas-Fernandez, Hong-Hoi Ting, Vasko Kramer, Malik E. Juweid, Felix M. Mottaghy

**Affiliations:** 1 Department of Nuclear Medicine, University of Bonn, Bonn, Germany; 2 Betaklinik Bonn, Bonn, Germany; 3 Berking Theranostics, Luruper Hauptstrasse 1, D-22547 Hamburg, Germany; 4 Nanomab Technology, 720 Centennial Court, Centennial Park, Elstree, WD63SY Borehamwood, UK; 5 Acrux Radiopharma Consulting SpA, Santiago, Chile; 6 Department of Radiology and Nuclear Medicine, University of Jordan, Amman, Jordan; 7 Department of Nuclear Medicine, University Hospital Aachen, Pauwelsstr. 30, D-52072 Aachen, Germany; 8 Department of Radiology and Nuclear Medicine, Maastricht University Medical Center (MUMC+), Maastricht, The Netherlands

In 1906, Paul Ehrlich -Nobel Laureate and one of the fathers of immunology- postulated that antibodies might be useful agents for specific targeting of diseases and coined the term “magic bullets” [1]. Specific antibodies were used for checking the blood of patients with suspected or proven malignant tumors and their recurrence. In the 1980’s and 1990’s, radiolabelled tumor marker specific antibodies or their Fab or F(ab‘)2 fragments were used for the in vivo detection of respective malignancies [2] employing planar scintigraphy and SPECT but were later substituted by dedicated PET probes and/or the improved detection rate using PET/CT.

The discovery of single domain antibodies, now known as nanobodies dates back to 1993. They were first described as a specific feature of the immune system of camels [3]. In this species, antibodies consisting of only heavy chains instead of hitherto known antibodies with 2 heavy and 2 light chains were identified. Similar heavy chain antibodies (nanobodies) were later also found in sharks. The low molecular mass (12–15 kDa) of the nanobodies allows penetration deep into the tumor, and those unbound to the tumor are quickly excreted by the kidneys [4].

Specific nanobodies are screened and selected from a phage, bacterial or yeast display library generated from peripheral blood lymphoctyes of Camelids immunised against its targeting antigen [5] and are useful in various medical applications:

Nanobodies specific to respective cell-surface biomarkers may be labelled with gamma, beta or alpha emitters for molecular imaging and theranostics [4]. Data on preclinical screening have already been published by Vaneycken et al. [6] in 2011 concerning anti-HER2 nanobodies in breast cancer. Clinical data on Tc-99 m labelled HER2 nanobodies in diverse cancer types were later published by Altunay et al. [7] and on Ga-68 HER2 in breast cancer by Gondry et al. [8]. Preclinical studies by Wong et al. [5] using Tc-99 m NM-01 showed uptake in PD-L1 bearing cancer cell-lines. Dekempeneer et al. [9] performed preclinical studies using anti-HER2 nanobodies labelled with Astatine-211 for radionuclide therapy. The excellent 2021 review article by Bao et al. in the EJNMMI Research [4] details the various applications of nanobodies for molecular imaging and therapy, including imaging of cancer, atherosclerosis, rheumatoid arthritis and hepatic inflammatory disorders. The advantages of nanobody-based ligands for therapy when combined with I-131, Lu-177 and Ac-225 are their fast blood clearance and suitability for conjugation while the high renal uptake with potential nephrotoxicity is the main disadvantage; the latter can be partially countered by co-injecting with cationic amino acids [4]. Overall, the data suggest that camelid nanobodies are likely to play an increasingly important role in nuclear medicine.

Li et al. [10, 11] have shown that camelid nanobodies with a basic isoelectric point are able to readily transmigrate across the blood brain barrier (BBB) and, when targeting specific intracerebral epitopes, may potentially help cure Alzheimers disease (AD). They used novel nanobodies specifically recognizing extracellular amyloid deposits and intracellular tau neurofibrillary tangles, the two core lesions of AD. Utilizing this approach in vivo real-time two-photon microscopy was able to show gradual extravasation of the nanobodies across the BBB, diffusion into the parenchyma, and labelling of amyloid deposits and neurofibrillary tangles [11]. These nanobodies may potentially be able to dissolve the amyloid plaques and, when radiolabelled with PET and/or SPECT tracers be used in the diagnosis and posttherapy follow-up of AD.

In 2024, Rizk et al. published a review on the significance of camelid nanobodies in the fight against infectious diseases [12]. The first approved nanobody (2018) for a therapeutic indication was caplacizumab in patients with acquired thrombocytopenic purpura. “Nature‘s tiny weapons” will likely help improve therapy of infections with multiresistant pathogens. Recently a CD45 targeting nanobody was introduced for the quantitative as well as longitudinal use for imaging inflammation. In the preclinical setting this new probe outperforms FDG [13].

In summary, camelid nanobodies are already widening the field of medicine, including nuclear medicine and will likely become an integral part of molecular imaging and theranostics.
